# Efficacy of pipeline embolization device vs. traditional coils in embolization of intracranial aneurysms: A systematic review and meta-analysis

**DOI:** 10.3389/fneur.2022.978602

**Published:** 2022-09-29

**Authors:** Wei Li, Zaixing Xiao, Kaixuan Zhao, Shijie Yang, Yichuan Zhang, Bin Li, Yu Zhou, Yong Ma, Erqing Chai

**Affiliations:** ^1^The First Clinical Medical College of Gansu University of Chinese Medicine, Lanzhou, China; ^2^Cerebrovascular Disease Center of Gansu Provincial People's Hospital, Lanzhou, China; ^3^Key Laboratory of Cerebrovascular Diseases in Gansu Province, Lanzhou, China; ^4^The First Clinical Medical College of Lanzhou University, Lanzhou, China

**Keywords:** PED vs. traditional coils efficacy pipeline embolization device (PED), traditional coils, intracranial aneurysm, comparative efficacy, systematic review, meta-analysis

## Abstract

**Introduction:**

In recent years, the Pipeline embolization device (PED) has been widely used in the embolization of intracranial aneurysms, but there are some inconsistent findings on whether its efficacy and safety are superior to those of traditional coils embolization (coils alone, stent-assisted coils and balloon-assisted coils). The purpose of this meta-analysis was to evaluate the safety and efficacy of PED in intracranial aneurysm embolization by comparing with traditional coils.

**Methods:**

We systematically searched PubMed, Embase, Web of Science, and The Cochrane Library databases for randomized controlled trials and observational studies (case-control studies and cohort studies) comparing the efficacy of PED with traditional coils in intracranial aneurysm embolization published before April 1, 2022. The endpoints observed in this meta-analysis were procedure-related intracranial hemorrhage, procedure-related intracranial ischemia, other procedure-related complications (e.g., aneurysm rupture, neurological impairment, etc.), retreatment rate, complete occlusion (100%) of the aneurysm at the last follow-up, and favorable functional outcome (MRS ≤ 2).

**Results:**

A total of 10 studies with a total of 1,400 patients (PED group: 576 and Traditional coils: 824) were included in this meta-analysis. A comprehensive analysis of the included literature showed that the PED group had a higher rate of complete aneurysm occlusion [OR = 2.62, 95% Cl (1.94, 3.55), *p* < 0.00001] and Lower re-treatment rate [OR = 0.20, 95% Cl (0.12, 0.34 *p* < 0.00001)] compared with the traditional coil embolization group at the last follow-up. In terms of procedure-related intracranial hemorrhage [OR = 3.04, 95% Cl (1.08, 8.57), *p* = 0.04] and other procedure-related complications [OR = 2.91, 95% Cl (1.48, 5.57), *p* = 0.002], the incidence of PED was higher than that of the traditional coil embolization group. Moreover, in terms of favorable functional outcome [OR = 0.4, 95% Cl (0.22, 0.71), *p* = 0.002] of patients at the last follow-up, the PED group was lower than the traditional coil embolization group. There was no statistically significant between the two groups in terms of surgery-related intracranial ischemia complications [OR = 0.88, 95% Cl (0.47, 1.64), *p* = 0.68].

**Conclusion:**

PED had higher rates of complete aneurysm occlusion and lower rates of aneurysm retreatment compared with traditional coils, but traditional coils was superior to the PED group in terms of procedure-related intracranial hemorrhage complication and other procedure-related complications (aneurysm rupture, neurological impairment), and favorable functional outcome (mRS ≤ 2). This result still needs to be further confirmed by additional large-sample, multicenter, prospective randomized controlled trials.

**Systematic review registration:**

https://www.crd.york.ac.uk/PROSPERO/, identifier: CRD42022325673.

## Introduction

Ruptured intracranial aneurysms can lead to severe subarachnoid hemorrhage and threaten patients' life (1). In recent years, researchers have focused on finding treatments to reduce the morbidity and mortality of intracranial aneurysms. With the rapid development of endovascular techniques, endovascular treatment provides new treatment options for aneurysms and has become the preferred modality for the treatment of certain intracranial aneurysms.

Pure coil embolization is mainly used for small and uncomplicated aneurysms. Stent-assisted spring coil (SAC) is an alternative technique for the treatment of giant, wide-necked, and spindle-shaped aneurysms that have failed to respond to pure coil embolization therapy, where the propped-up stent prevents the coil from entering the aneurysm-carrying artery (2). However, this traditional coils embolization technique has significant treatment limitations, and numerous studies have found that ~12–14.5% of aneurysms after coils embolization therapy are recanalized after occlusion, increasing the risk of aneurysm re-rupture (3, 4). The pipeline embolization device (PED; Covidien, Medtronic) was the first vascular diversion device approved for the treatment of large or large wide carotid aneurysms from the carotid to the superior segment of the pituitary in the internal carotid artery (ICA).The PED diverts the blood flow into the aneurysm, leading to thrombosis in the interior of the aneurysm lumen, and subsequently reconstructs the lumen of the aneurysm-carrying artery by endothelialization of the stent (5) to achieve the purpose of aneurysm occlusion. With the development of PED technology, its clinical indicators are gradually expanding (the so-called “out-of-indication” use), and it is necessary to compare PED with the traditional coils embolization technique to assess safety and efficacy.

However, the most appropriate strategy for the endovascular treatment of aneurysms depends mainly on clinical factors and the aneurysm's anatomical characteristics. The choice of the best endovascular approach for treatment remains to be determined. In several studies compared with conventional coil embolization, PED treatment significantly increases the rate of aneurysm occlusion and decreases the rate of retreatment and complications (6, 7). However, there are also studies showing that PED treatment is not as safe and effective as assumed (8). In this study, a meta-analysis was performed to evaluate the safety and efficacy of PED in intracranial aneurysm embolization through a randomized controlled trial and an observational study comparing the efficacy of PED with traditional coils in intracranial aneurysm embolization.

## Methods

### Search strategy

This meta-analysis was performed according to the PRISMA guidelines. We systematically searched PubMed, Embase, Web of Science, and The Cochrane Library databases for randomized controlled trials and observational studies (case-control studies and cohort studies) comparing the efficacy of PED with traditional coils in embolization of intracranial aneurysms published before April 1, 2022. A literature search was conducted independently by two investigators, and we used a combination of the following terms: Intracranial Aneurysm (Mesh), Aneurysm, Anterior Communicating Artery, Aneurysm, Basilar Artery, Aneurysm, Middle Cerebral Artery, Aneurysm, Posterior Cerebral Artery, Berry Aneurysm, Brain Aneurysm, Cerebral Aneurysm, Giant Intracranial Aneurysm Mycotic Aneurysm, Intracranial, Aneurysm, Anterior Cerebral Artery, Aneurysm, Posterior Communicating Artery, Pipeline embolization device, Flow diverter device, PED, Pipeline Flex, primary coil, balloon-assisted coiling, stent-assisted coiling. References generated from these searches were imported into the reference manager EndNote X9.3.1 (Thompson Reuters, Philadelphia, PA) and duplicate references were removed. Then, journal article titles and abstracts were systematically screened by two researchers independently according to the following inclusion and exclusion criteria. This meta-analysis has been registered in PROSPERO (ID: CRD42022325673).

### Inclusion criteria

(1) Patients with confirmed intracranial aneurysms (ruptured and unruptured intracranial aneurysms) (2) Vascular treatment: with PED and traditional coils embolization (coils alone, stent-assisted coils, balloon-assisted coils) (3) Data for two treatment groups can be clearly provided in the literature: the PED treatment group and the traditional coils embolization group (4) Randomized controlled trials and observational studies (case-control studies and cohort studies).

### Exclusion criteria

(1) unpublished studies, conference abstracts, letters, reviews, correspondence, and animal studies; (2) studies with duplicate or overlapping data; (3) lack of outcome data outside of hospitalization; and (4) literature that did not provide data for both treatment groups: the PED treatment group and the traditional coils embolization group (5) case series of < 10 patients for both.

### Antiplatelet therapy strategy

Prior to PED or stent-assisted coil embolization, patients were given a loading dose of 325–650 mg aspirin and 600 mg clopidogrel as antiplatelet therapy for patients with acute ruptured aneurysms. For non-emergency patients, 1–2 weeks before treatment, patients were started on daily aspirin (ASA) 100–325 mg and clopidogrel 75 mg antiplatelet aggregation. Use light transmittance aggregometr (LTA) or thromboelastography (TEG) to perform platelet function tests, and determine whether to adjust the drug dose or replace antiplatelet drugs according to the test results. Dual antiplatelet therapy was generally continued for 6 months after device placement, followed by aspirin indefinitely (5–7). The choice of oral antiplatelet drug timing and aspirin dose for treatment initiation varies by patient ethnicity and other differences and is selected according to national guidelines.

### Data extraction and efficacy metrics

Data for each eligible literature were extracted independently by 2 investigators, and any disagreements were resolved by discussion and consultation with a 3rd senior neurosurgeon. Basic information such as first author's name, study design, sample size, mean age, sex ratio, size of the aneurysm, width of the aneurysm neck, location of the aneurysm, and endovascular treatment modality were extracted using a pre-developed form. The main indicators analyzed: procedure-related intracranial hemorrhage, procedure-related intracranial ischemia, other procedure-related complications (e.g., aneurysm rupture, impaired neurological function, etc.), retreatment rate, complete occlusion (100%) of the aneurysm at the last follow-up, and favorable functional outcome (MRS ≤ 2).

### Literature quality assessment

Each of the two trained researchers read all the titles and abstracts of the literature, firstly screened out the literature that clearly did not meet the inclusion criteria, and then read the full text of the literature to initially identify the literature that could be included in the study. Finally, the screening results of the two researchers were cross-checked, and the two evaluators discussed the questionable literature and combined the third-party opinions to decide whether to include it or not. The quality of randomized controlled trials was evaluated using the Cochrane Risk of Bias tool, and the quality of observational studies was evaluated using the Newcastle-Ottawa Scale (NOS).

### Statistics analysis

Statistical analyses were performed using Review Manager (v.5.3), and differences were considered statistically significant at *P* ≤ 0.05 if not explicitly stated. We calculated the odds ratio (OR) of categorical variables using a random-effects model, and heterogeneity was evaluated using chi-square tests and *I*^2^ tests, and we considered data to be significantly heterogeneous when *I*^2^ >50%, and we performed meta-analysis using a random-effects model, otherwise, a fixed-effects model was performed. Sensitivity analyses were performed by omitting studies one by one to assess the effect of each study on the overall outcome. Symmetry was assessed using Begg's and Egger's tests, and significant publication bias was defined as *p* < 0.1, and publication bias was assessed with sensitivity analysis using STATA (v.12).

## Results

### Search results and selection of research subjects

**Figure 1:**
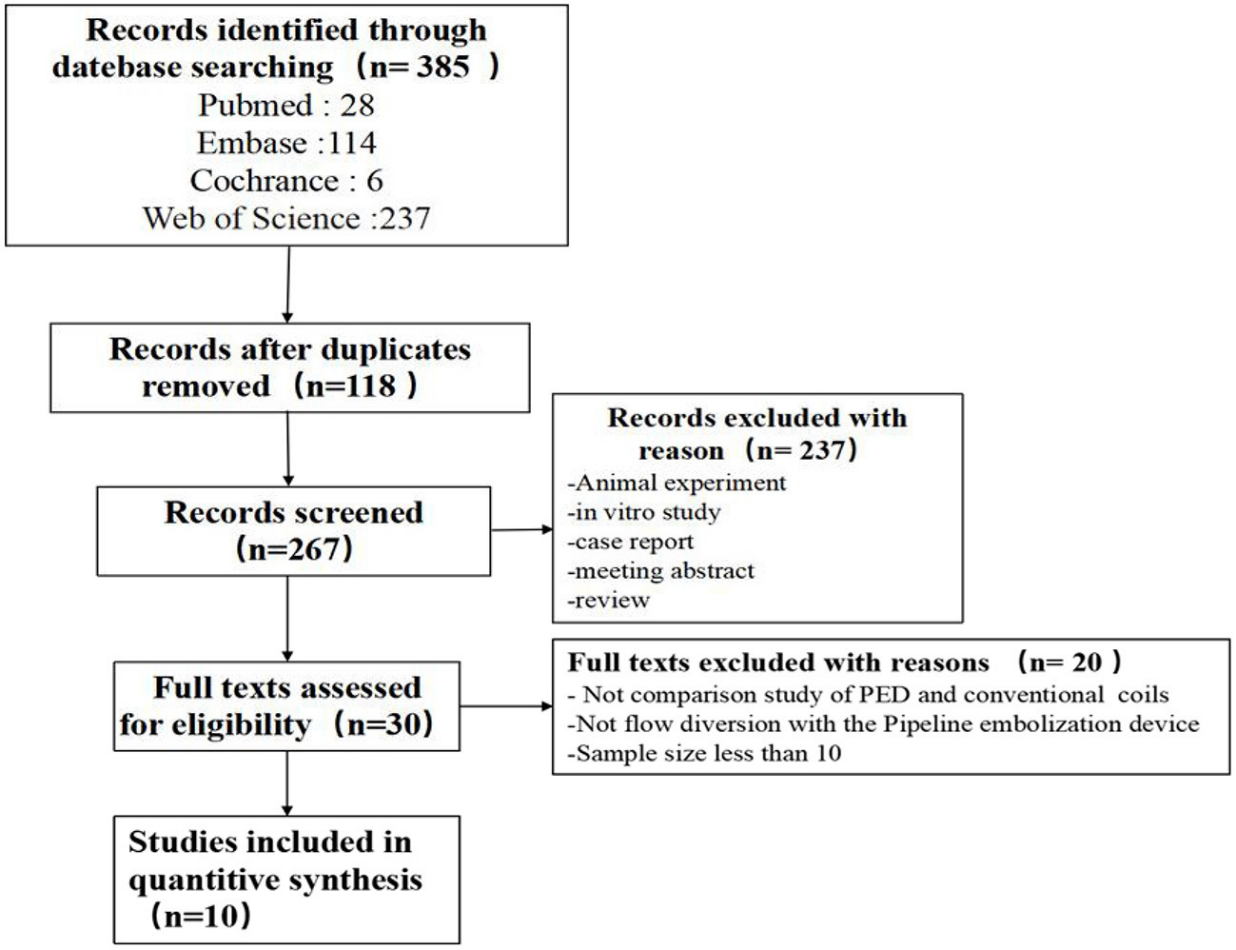
Flow chart of the search and inclusion of literature.

Searching from the database identified 385 articles (Pubmed: 28, Embase: 114, Cochrance: 6, Web of Science: 237), of which 118 duplicates were excluded. The titles and abstracts of the shortlisted articles were reviewed and excluded An additional 237 papers were reviewed, and the remaining 30 papers were read in detail to determine whether they met the inclusion/exclusion criteria. Ultimately, 10 eligible papers were included in this meta-analysis (7, 9–17) (shown in Figure 1).

### Basic characteristics of the research object

One thousand four hundred patients from 10 (7, 9–17) studies (0 randomized controlled trials and 10 observational studies) were included in this study, of whom 576 were treated with PED and 824 with traditional coils embolization. Demographic characteristics and details regarding the type of literature included in the study are shown in Table 1.

**Table 1:** Baseline characteristics of the included studies.

**References**	**Study design**	**Sample size**		**Aneurysm size (mm)**		**Aneurysm neck size (mm)**		**Aneurysm location**	**Mean age, years (P/T)**	**Gender (M/F)**		**Endovascular therapy**
		**P**	**T**	**P**	**T**	**P**	**T**			**P**	**T**	
Chalouhi et al. (7)	Observational	40	120	14.9 ± 4.7	14.9 ± 5.9	5.0 ± 1.2	4.9 ± 1.7	OA, VA, MCA, PcomA, cavernous, paraclinoid, petrous	60.7/60.3	7/33	17/103	PED, coiling, SAC, BAC
Di Maria et al. (9)	Observational	77	61	8.7 + 6.3	6.7 + 3.6	Na		Carotid-ophthalmic aneurysms	49.7/49.2	17/60	10/51	PED, coiling, SAC, BAC
Zanaty et al. (10)	Observational	51	106	16.75	14.27	Na		Carotid cavernous aneurysms	63.0/60.42	4/47	7/99	PED, coiling, SAC
Adeeb et al. (11)	Observational	106	62	6.4	7.1	4	5.1	Ophthalmic segment aneurysms	57/57	8/98	1/61	PED, SAC
Chalouhi et al. (12)	Observational	40	40	6.3 ± 2.7	6.3 ± 2.8	Na		Paraclinoid, PcomA, OA, carotid cave	54.8/54.9	4/40	4/40	PED, coiling, SAC, BAC
Zhang et al. (13)	Observational	55	300	4.3 ± 1.4	4.0 ± 1.3	Na		Cavernous, OA, paraclinoidal	54.1/53.4	9/55	50/250	PED, coiling, SAC
Enriquez-Marulanda et al. (14)	Observational	21	17	4.9	8.6	Na		Communicating segment ICA	61/58	4/17	2/15	PED, SAC
Zhang et al. (15)	Observational	30	64	11	11.6	Na		Intradural vertebral artery aneurysms	51/53	24/6	57/7	PED, SAC
Salem et al. (16)	Observational	135	30	4.9	5.2	Na		ICA, carotid bifurcation	58/60.5	22/135	5/25	PED, SAC
Suzuki et al. (17)	Observational	21	24	12.3 ± 3.6	12.9 ± 3.2	6.1 ± 1.8	6.9 ± 2.5	Paraclinoid aneurysms	59/60.6	4/17	7/17	PED, coiling, SAC, BAC

P, pipeline embolization device (PED); T, traditional coils embolism; OA, ophthalmic segment; VA, vertebrobasilar; MCA, middle cerebral artery; PcomA, middle cerebral artery; ICA, internal carotid artery.

### Quality evaluation of the included literature

A total of 10 (7, 9–17) studies were included, and all 10 studies were observational, using NOS quality assessment of non-randomized controlled trials (Supplementary Table 1). In conclusion, the quality scores of the included literature were high, describing the selection of the study population and comparability between groups.

### PED vs. traditional coils for efficacy

#### Procedure-related intracranial hemorrhage

**Figure 2:**
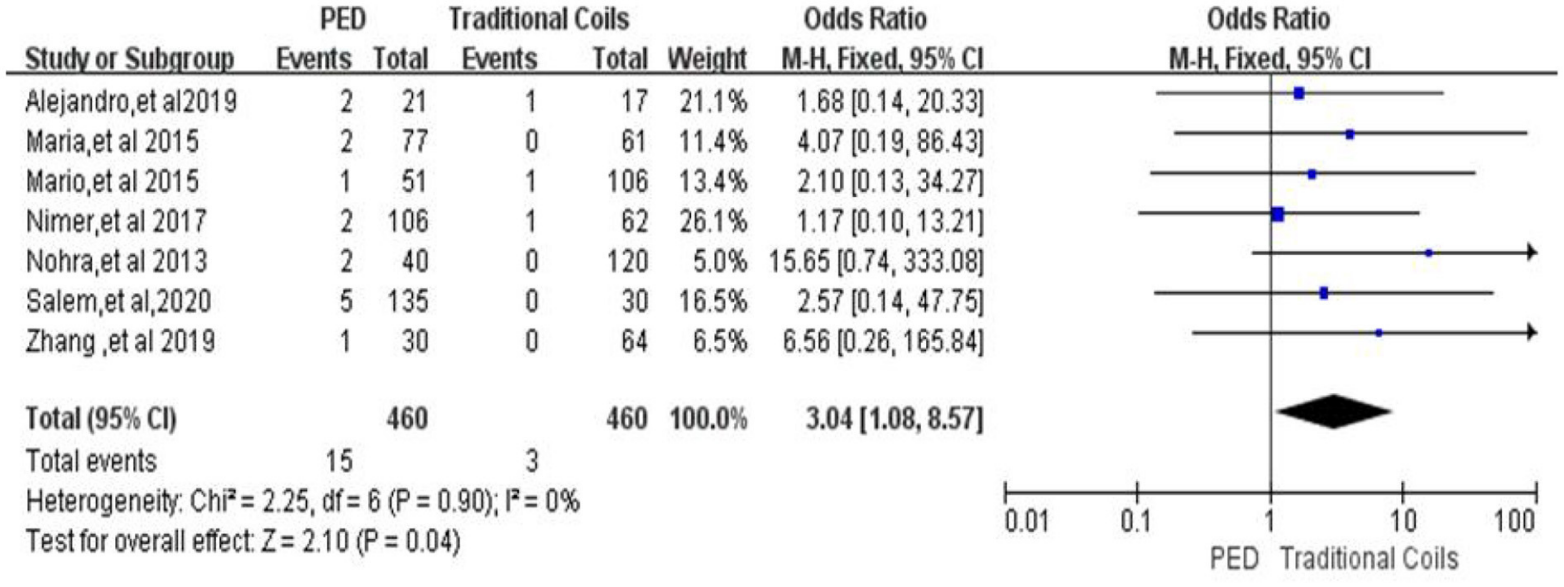
Forest plot and meta-analysis of procedure-related intracranial hemorrhage.

In the evaluation of procedure-related intracranial hemorrhage, a total of seven (7, 9–11, 14–16) studies were included, with a total of 460 patients in the PED group with 15 (3.3%, 15/460) patients with procedure-related intracranial hemorrhage and a total of 460 patients in the traditional coils group with three (0.7%, 3/460) patients with procedure-related intracranial hemorrhage, with low heterogeneity (*I*^2^ = 0%, *P* = 0.90), so a fixed-effects model was used. The incidence of Procedure-related intracranial hemorrhage was higher in the PED group than in the conventional coil embolization group, with a statistically significant difference between the two groups [OR = 3.04, 95% Cl (1.08, 8.57), *p* = 0.04; shown in Figure 2].

#### Procedure-related intracranial ischemia

**Figure 3:**
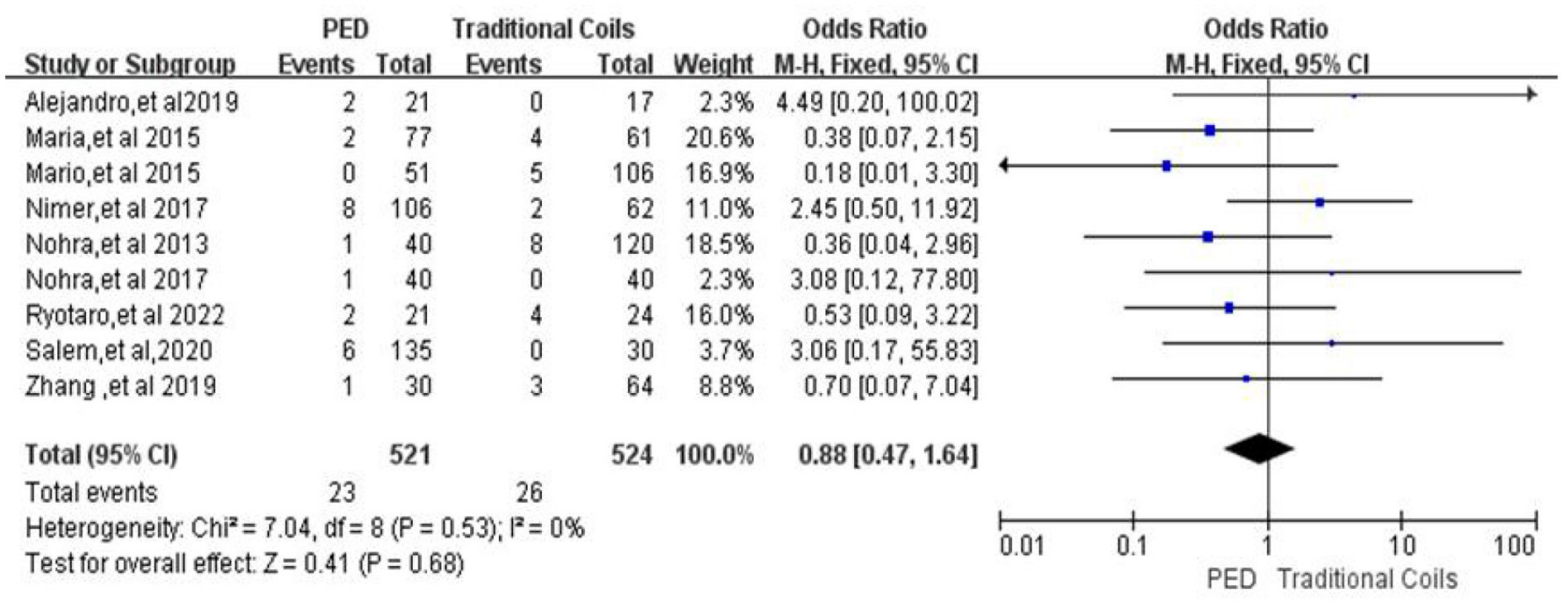
Forest plot and meta-analysis of procedure-related intracranial ischemia.

In the evaluation of procedure-related intracranial ischemia, a total of nine (7, 9–12, 14–17) studies were included, with a total of 521 patients in the PED group and 23 (4.4%, 23/521) patients with procedure-related intracranial ischemia, and a total of 524 patients in the traditional coils group and 26 (4.9%, 26/524) patients with procedure-related intracranial ischemia, with low heterogeneity (*I*^2^ = 0%, *P* = 0.53), so a fixed-effects model was used. There was no statistically significant difference between the two groups in terms of Procedure-related intracranial ischemia [OR = 0.88, 95% Cl (0.47, 1.64), *p* = 0.68; shown in Figure 3].

#### Other procedure-related complications

**Figure 4:**
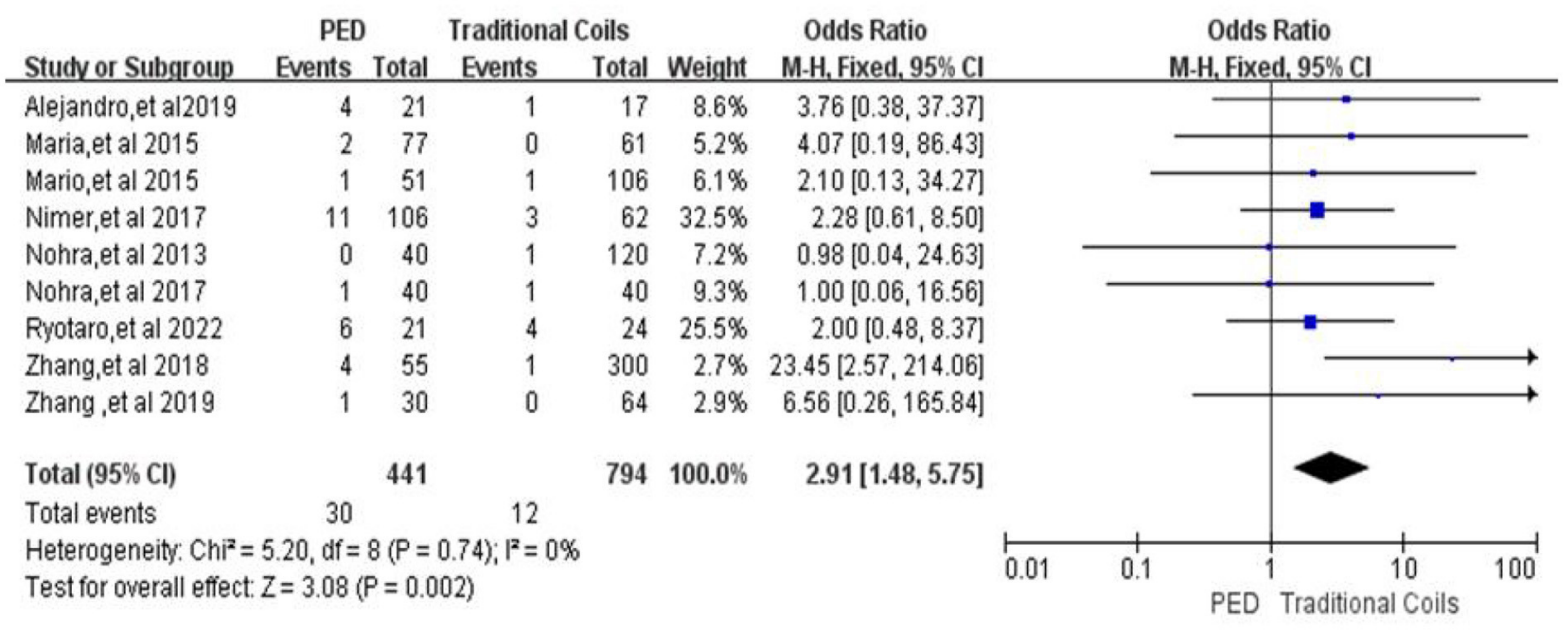
Forest plot and meta-analysis of other procedure-related complications.

In the evaluation of other procedure-related complications, a total of nine (7, 9–15, 17) studies included a total of 441 patients in the PED group with 30 (6.8%, 30/441) patients with other procedure-related complications and a total of 794 patients in the traditional coils group with 12 (1.5%, 12/794) patients with other procedure-related complications, with low heterogeneity (*I*^2^ = 0%, *P* = 0.74), so a fixed-effects model was used. In terms of other procedure-related complications (aneurysm rupture, neurological deficit), the PED group had a higher incidence than the traditional coil embolization group, and there was a statistically significant difference between the two groups [OR = 2.91, 95% Cl (1.48, 5.57), *p* = 0.002; shown in Figure 4].

#### Aneurysm retreatment rate

**Figure 5:**
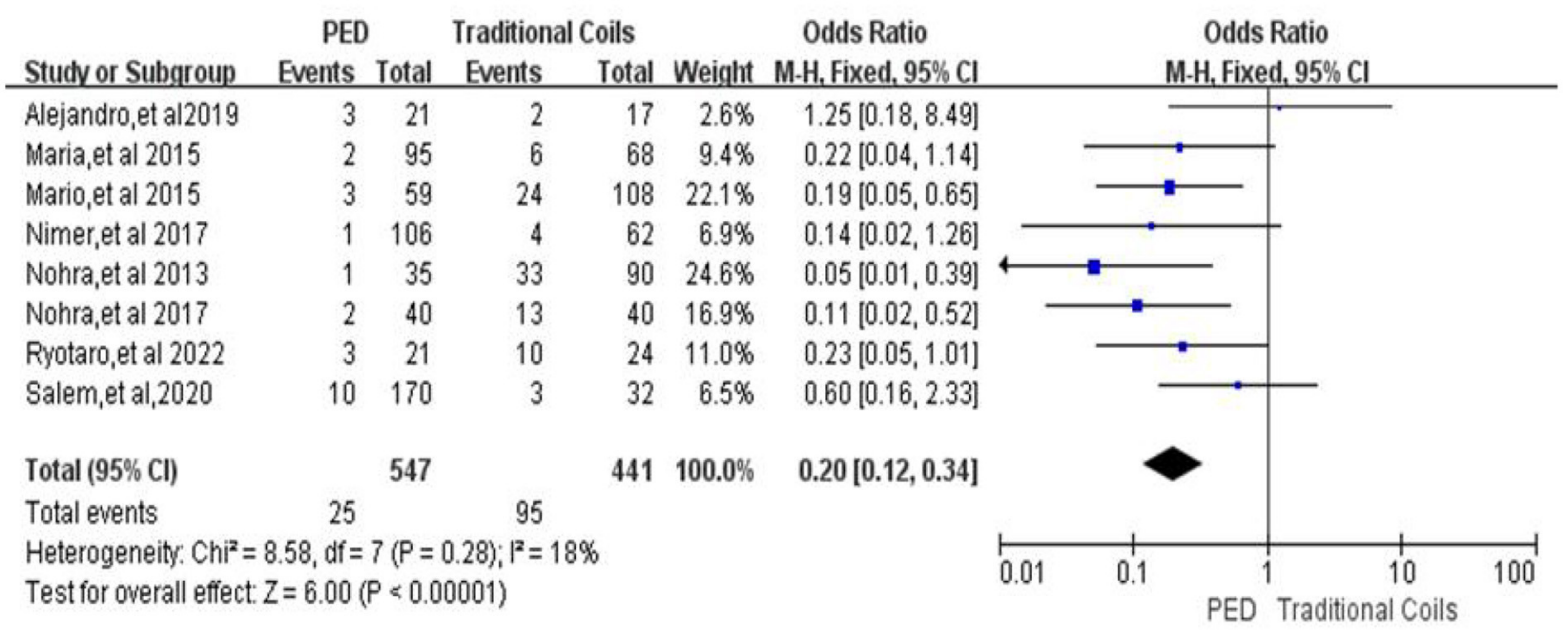
Forest plot and meta-analysis of aneurysm retreatment rate.

Aneurysm retreatment rates from a total of 998 intracranial aneurysms included in eight studies (7, 9–12, 14, 16, 17), heterogeneous (*p* = 0.28, *I*^2^ = 18%), using a fixed effects model, with a retreatment rate of 4.6% (25/547) in the PED group and 21.5% (95/441) in the traditional coils group, using PED compared to traditional coils had a lower retreatment rate, with a statistically significant difference between the two [OR = 0.20, 95% Cl (0.12, 0.34), *p* < 0.00001; shown in Figure 5].

#### Favorable functional outcome of patients at last follow-up (MRS ≤ 2)

**Figure 6:**
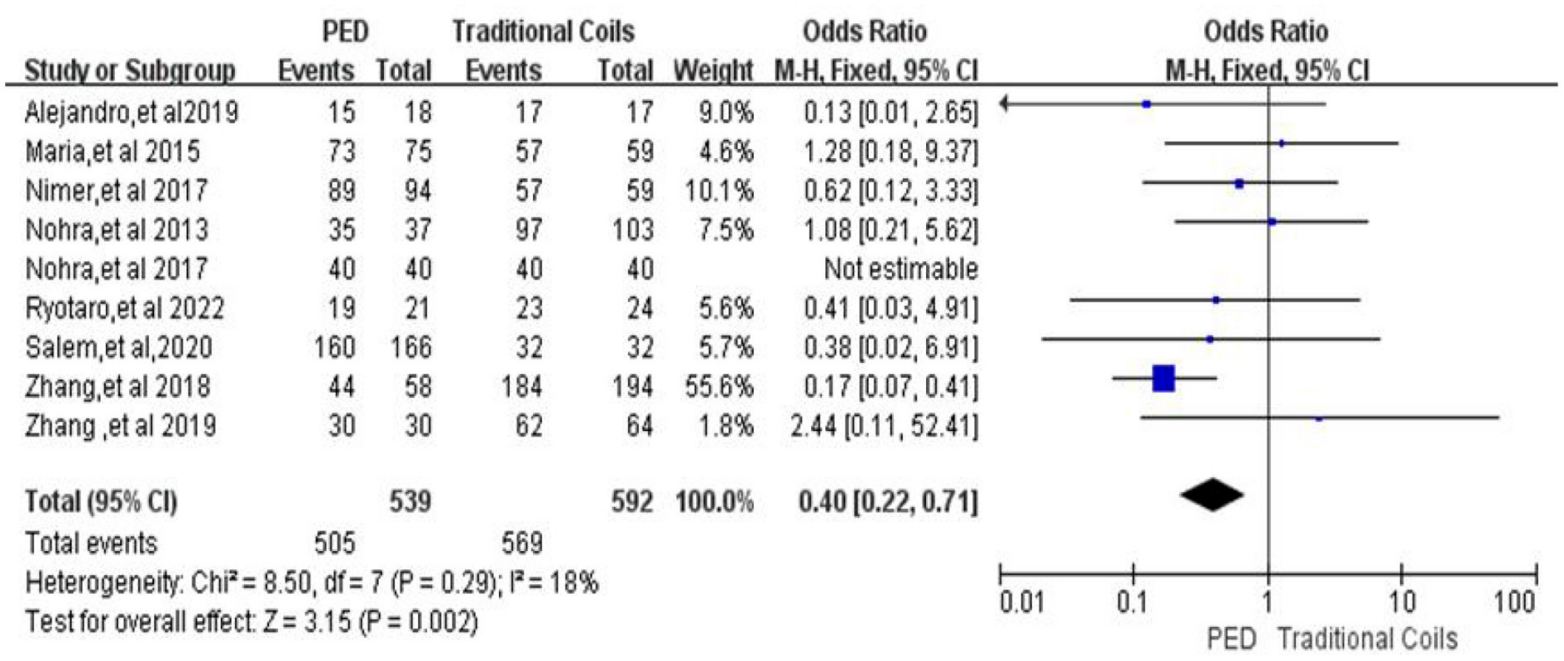
Forest plot and meta-analysis of favorable functional outcome of patients at last follow-up.

In the evaluation of favorable functional outcome of patients at follow-up, a total of nine (7, 9, 11–17) studies were included, with 539 patients in the PED group with a MRS 0–2 score of 505 (93.7%, 505/539) and 592 patients in the traditional coils group with a MRS ≤ 2 score of 569 (96.1%, 569/592), with low heterogeneity (*I*^2^ = 18%, *P* = 0.29), using a fixed-effects model. Compared with the traditional coil embolization group, the PED group had fewer patients with MRS ≤ 2 at last follow-up, and the difference between the two was statistically significant [OR = 0.4, 95% Cl (0.22, 0.71), *p* = 0.002; shown in Figure 6].

#### Complete occlusion rate (100%) of aneurysm in patients at last follow-up

**Figure 7:**
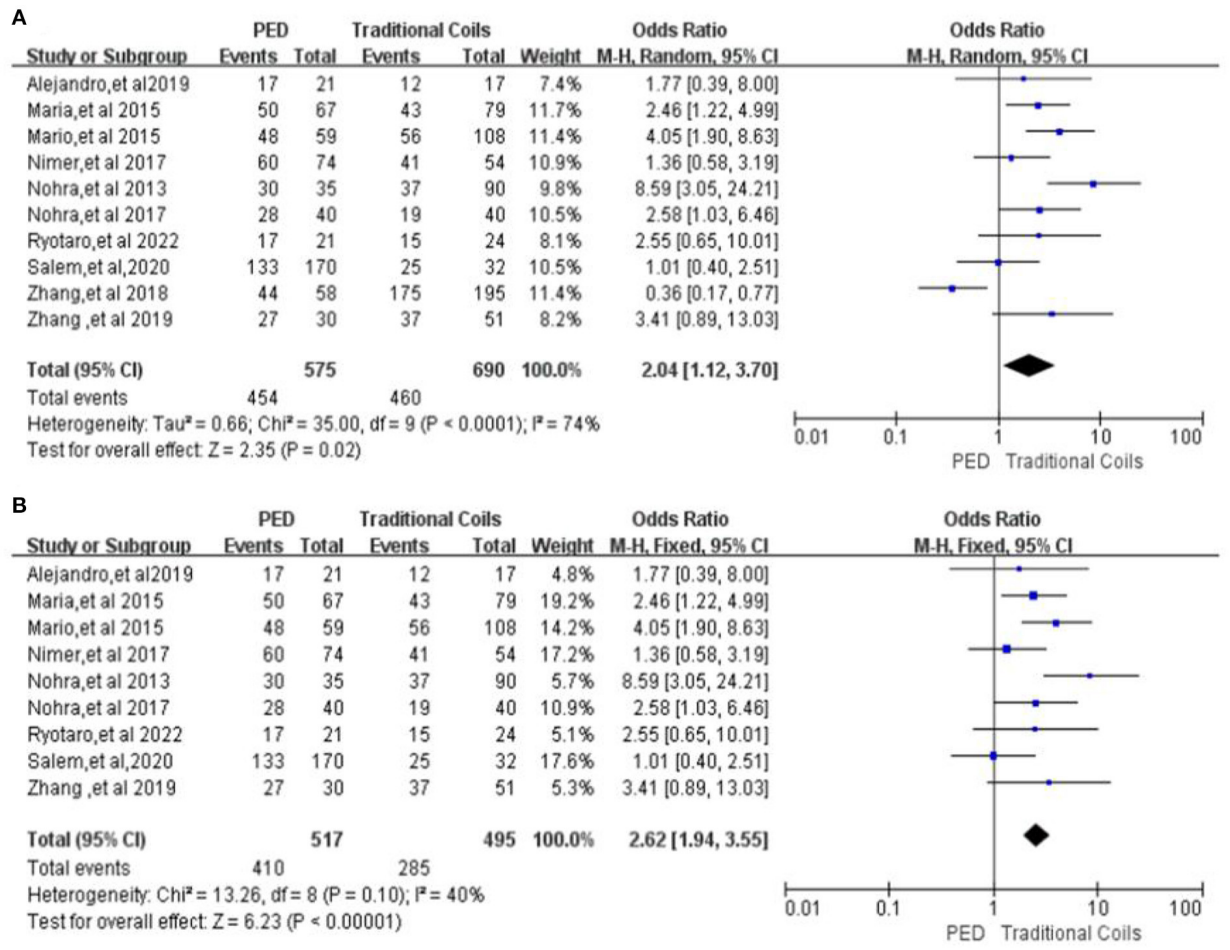
**(A)** Forest plot and meta-analysis of complete occlusion rate (100%) of aneurysm in patients at last follow-up. **(B)** Forest plot and meta-analysis of Complete occlusion rate (100%) of aneurysm in patients at last follow-up after excluding the study by Zhang et al. to reduce heterogeneity.

A total of 10 studies were included in the comparison of patients with complete occlusion of aneurysms in the two groups at last follow-up (7, 9–17), with high heterogeneity (*P* < 0.0001, *I*^2^ = 74%), with 575 aneurysms followed in the PED group and 454 complete occlusion, with a complete occlusion rate of 78.96%, and 690 aneurysms followed in the traditional coils group and 460 complete occlusion, with a complete occlusion rate was 66.67%, and PED had a higher occlusion rate compared to traditional coils, with a statistically significant difference between the two [OR = 2.04, 95% Cl (1.12, 3.70), *p* = 0.02, shown in Figure 7A]. After excluding the study by Zhang et al. (13), the heterogeneity of this analysis was significantly lower (*I*^2^ = 40%, *p* = 0.1), with complete occlusion rates of 79.3% (410/517) in the PED group and 57.6% (285/495) in the traditional coils group, without affecting the final outcome [OR = 2.62, 95% Cl (1.94, 3.55), *p* < 0.00001, shown in Figure 7B].

### Sensitivity analysis and publication bias

In this meta -analysis, the results of the sensitivity analysis for effectiveness and safety were consistent with the results of the combined analysis; we used the Begg's and Egger's tests to assess the effect of publication bias, and the funnel plots were both symmetrical, with no significant evidence of publication bias.

## Discussion

PED is the earliest blood flow diverting device used for intracranial aneurysm embolization, and it was mainly used to treat large and giant wide-necked aneurysms of the internal carotid artery in the early stage. With the maturation of PED treatment technology in recent years, PED treatment has also started to be used super-indicated for small aneurysms, but the feasibility and advantages of the treatment are still controversial. The traditional coils embolization treatment modality has shown acceptable safety and effectiveness (18, 19), which makes it necessary to compare PED with traditional coils (coils alone, stent-assisted coils, balloon-assisted coils) safety and efficacy in the treatment of intracranial aneurysms. A total of 10 studies comparing the two treatment modalities involving 1,400 patients were included in this meta-analysis. After a comprehensive analysis it was shown that the PED group had a lower retreatment rate and a higher rate of complete aneurysm occlusion (100%) compared to the traditional coils embolization group. The traditional coils embolization group was superior to the PED group in terms of procedure-related intracranial hemorrhage, other procedure-related complications (aneurysm rupture, neurological impairment, etc.), and favorable functional outcome at last follow-up (MRS ≤ 2), but no significant differences were seen between the two groups in terms of procedure-related intracranial ischemic complications.

Endovascular therapy is now the key treatment for most different types of intracranial aneurysms. Coil embolization is traditionally one of the most popular treatment modalities and is primarily indicated for the treatment of small (< 10 mm), unruptured and morphologically simple anterior circulation intracranial aneurysms. Stent-assisted coil embolization is based on simple coil embolization to solve the problems of residual aneurysm neck and coil protruding into the parent artery through stent-assisted embolization, and can be used as the core of endothelial cell growth and aneurysm healing (20, 21). Compared with coil embolization alone, stent-assisted spring coil embolization has a higher rate of complete occlusion and a lower rate of recurrence (22). Although stent-assisted coils have wider indications and better efficacy than coils alone, there are still technical challenges, such as difficulty in passing microguidewires and microcatheters through the stent gap, stent misalignment, and incomplete coiling leading to residual aneurysm neck, making the persistence of aneurysm occlusion still a concern. The introduction of PED technology overcomes some of the technical challenges of conventional spring coil embolization. PED is a specialized shunt approved by the U.S. Food and Drug Administration (FAD) in 2011. It works to rebuild the parent artery, thereby Diverts blood flow away from the aneurysm, resulting in interruption and stagnation of blood flow within the aneurysm, subsequent thrombus formation, and occlusion of the aneurysm, while the vital arterial branches covered by the shunt remain open (23). The safety and efficacy of PEDs have also been confirmed in several recent series, but most of these series were not comparative studies with patients treated with traditional embolization strategies (6, 24, 25). In 2013, Crobeddu et al. (26) reported that in the 4 years since PEDs were first introduced, the use of SAC decreased from 14.7 to 6.9%. The reason why PED technology is welcomed by the majority of operators may be mainly due to its technical advantages. PED can avoid entering the aneurysm sac, thereby reducing the risk of iatrogenic rupture when placing the coil, especially for smaller aneurysms. In addition, multiple nearby aneurysms can be treated in a single operation, which can re-establish the Plastics the entire vessel, thereby preventing aneurysm recanalization and formation of new aneurysms in the context of dysplastic parent vessels.

Whether the safety and efficacy of PED treatment of intracranial aneurysms is superior to that of traditional coils embolization is controversial. The most appropriate strategy for aneurysm embolization depends largely on clinical factors and the anatomic characteristics of the aneurysm. Previous studies have found a 1–8.6% incidence of procedure-related complications and a 5–23% re-treatment rate for traditional coils embolization of intracranial aneurysms (27–29). In the study of this meta-analysis, the incidence of procedure-related intracranial hemorrhage in the traditional coils embolization group was found to be 0.7%, the incidence of intracranial ischemia was 4.9%, the incidence of other procedure-related complications was 1.5%, and the re-treatment rate was 21.5%, which is similar to the results of previous studies. In contrast, regarding PED treatment, previous studies have reported rates of 3.4–31.7% for neurosurgery-related complications and 0.9–15% for retreatment (11, 30, 31). In the study of this meta-analysis, the incidence of procedure-related intracranial hemorrhage was 3.3%, the incidence of intracranial ischemia was 4.4%, the incidence of other procedure-related complications was 6.8%, and in the treatment rate was 4.6%. The results of previous studies were also similar. Because of the sample size of the original study, this meta-analysis did not include separate subgroup analyses of aneurysm size and location. In terms of the overall outcome of aneurysm treatment, the traditional coils embolization group was superior to the PED group in terms of procedure-related complications. However, in another study conducted by Zhang et al. (32), a propensity score analysis was performed to compare the safety and efficacy of PED vs. SAC in large and giant aneurysms, and procedure-related complications were similar between the two groups. Alejandro et al. (14) also compared PED and SAC for the treatment of aneurysms located in the traffic segment of the internal carotid artery, and the results showed that procedure-related complications were not significant between the two groups. This is inconsistent with our findings. We speculate that the main reason is that the aneurysms studied in this meta-analysis originate from blood vessels in various parts of the brain, and the sizes are different, which affects the consistency of the results. But we cannot ignore the unique complications of PED itself, such as delayed migration of the device, distal parenchymal hemorrhage, aneurysm rupture due to aneurysm wall degeneration or endoleakage (33–35). Large samples and randomized trials are still needed to validate for surgical complications. As for the re-treatment rate, our findings are consistent with those of previous studies, with the PED group was significantly better than the traditional coil embolization group (12, 17).

The rate of complete aneurysm occlusion during post-operative aneurysm follow-up is a key observation in the course of aneurysm treatment. In 2013, a matched study comparing PEDs and traditional coils for intracranial aneurysms found significantly higher occlusion rates for PED-treated aneurysms (86 vs. 41%) (7). Several single-center and multicenter studies have also demonstrated a higher rate of complete occlusion of intracranial arteries treated with PEDs compared with traditional aneurysm embolization strategies (7, 36). In the Di Maria et al. (9) comparative study found that the occlusion rate was also significantly higher in the PED group than in the traditional coils embolization group at 12 months follow-up (85.3 vs. 54%). However, some studies (14) also found no difference in complete aneurysm occlusion between the PED and traditional coils embolization groups. The mean duration of follow-up was 10 months in the PED group and 23 months in the traditional coils embolization group in the studies included in this meta-analysis, and the rate of complete aneurysm occlusion was significantly higher in the PED group than in the traditional coil embolization group at the last follow-up, which is consistent with the results of some of the previous studies. With regard to favorable functional outcome at the follow-up, several comparative studies on PED vs. stent-assisted coil treatment of aneurysms found no difference in favorable functional outcome (mRS ≤ 2) between the two groups during follow-up (11, 13, 16). This meta-analysis study found that the traditional coils embolization group was superior to the PED group with regard to favorable functional outcome at the last follow-up of the patients. We speculate that this has a certain relationship with the incidence of surgery-related complications in patients, and adverse complications lead to permanent neurological damage in patients. PED technique may have different efficacy for aneurysms of different sizes and locations, but in terms of overall results, PED still has a significant advantage in terms of complete aneurysm occlusion and aneurysm retreatment.

### Limitations

In interpreting the results, some limitations should be highlighted. First, most of the included studies were non-randomized, selection bias is inevitable, and different sizes and sites of aneurysms can affect the validity of the findings. Secondly, not all studies had the data required to assess the efficacy of PED vs. conventional spring coil embolization studies. Third, the overall sample size of this study was small, which may have affected the results.

## Conclusion

PED had higher rates of complete aneurysm occlusion and lower rates of aneurysm retreatment compared with traditional coils embolization, but traditional coils embolization was superior to the PED group in terms of procedure-related intracranial hemorrhagic complications and other procedure-related complications (aneurysm rupture, neurological impairment), and favorable functional outcome (mRS ≤ 2) at the last follow-up. This result still needs to be further confirmed by additional large-sample, multicenter, prospective randomized controlled trials.

## Data availability statement

The original contributions presented in the study are included in the article/Supplementary material, further inquiries can be directed to the corresponding author/s.

## Author contributions

WL and ZX participated in the design of the study, collected and analyzed the data, and drafted and revised the manuscript. KZ, SY, YZha, BL, YZho, and YM analyzed the data, interpreted the results, and performed the statistical analysis. EC designed the study, supervised the study inclusion and data extraction, and revised the manuscript. All authors contributed to the article and approved the submitted version.

## Conflict of interest

The authors declare that the research was conducted in the absence of any commercial or financial relationships that could be construed as a potential conflict of interest.

## Publisher's note

All claims expressed in this article are solely those of the authors and do not necessarily represent those of their affiliated organizations, or those of the publisher, the editors and the reviewers. Any product that may be evaluated in this article, or claim that may be made by its manufacturer, is not guaranteed or endorsed by the publisher.
