# Supplementary material for: Efficacy of pipeline embolization device vs. traditional coils in embolization of intracranial aneurysms: A systematic review and meta-analysis
Source: Front Neurol. 2022 Sep 29;13:978602. doi: 10.3389/fneur.2022.978602 (PMC9558282; doi:10.3389/fneur.2022.978602)
Supplement: Supplementary file 1 [file Data_Sheet_1.docx]

**Supplementary:**

**Search Type:**

((((((((((((((((((((((((((((Intracranial Aneurysm[Title/Abstract])) OR (Aneurysms, Intracranial[Title/Abstract])) OR (Intracranial Aneurysms[Title/Abstract])) OR (Aneurysm, Intracranial[Title/Abstract])) OR (Aneurysm, Anterior Communicating Artery[Title/Abstract])) OR (Anterior Communicating Artery Aneurysm[Title/Abstract])) OR (Aneurysm, Basilar Artery[Title/Abstract])) OR (Aneurysms, Basilar Artery[Title/Abstract])) OR (Artery Aneurysm, Basilar[Title/Abstract])) OR (Basilar Artery Aneurysms[Title/Abstract])) OR (Basilar Artery Aneurysm[Title/Abstract])) OR (Aneurysm, Middle Cerebral Artery[Title/Abstract])) OR (Middle Cerebral Artery Aneurysm[Title/Abstract])) OR (Aneurysm, Posterior Cerebral Artery[Title/Abstract])) OR (Posterior Cerebral Artery Aneurysm[Title/Abstract])) OR (Berry Aneurysm[Title/Abstract])) OR (Aneurysm, Berry[Title/Abstract])) OR (Brain Aneurysm[Title/Abstract])) OR (Cerebral Aneurysm[Title/Abstract])) OR (Giant Intracranial Aneurysm[Title/Abstract])) OR (Mycotic Aneurysm, Intracranial[Title/Abstract])) OR (Aneurysm, Intracranial Mycotic[Title/Abstract])) OR (Aneurysm, Anterior Cerebral Artery[Title/Abstract])) OR (Anterior Cerebral Artery Aneurysm[Title/Abstract])) OR (Aneurysm, Posterior Communicating Artery[Title/Abstract])) OR (Posterior Communicating Artery Aneurysm[Title/Abstract])) AND (((((Pipeline embolization device[Title/Abstract])) OR (Flow diverter device[Title/Abstract])) OR (PED[Title/Abstract])) OR (Pipeline Flex[Title/Abstract]))) AND ((((primary coil[Title/Abstract])) OR (balloon-assisted coiling[Title/Abstract])) OR (stent-assisted coiling[Title/Abstract]))

Supplementary Table1: Inclusion of observational studies literature NOS evaluation scales

| Study | Selection | | | | Comparability  Control for important factor | Exposure | | | Scores |
| --- | --- | --- | --- | --- | --- | --- | --- | --- | --- |
|  | Adequate definition of cases | Representativeness of the cases | Selection of controls | Definition of controls |  | Ascertainment of exposure | Same method Of ascertainment for Cases And Controls | Non-response rate |  |
| Nohra, et al 2013 [7] | 1 | 1 | 0 | 1 | 2 | 1 | 1 | 1 | 8 |
| Maria,et al  2015[9] | 1 | 1 | 0 | 1 | 2 | 1 | 1 | 1 | 8 |
| Mario,et al  2015[10] | 1 | 1 | 0 | 1 | 2 | 1 | 1 | 1 | 8 |
| Nimer,et al  2017[11] | 1 | 1 | 0 | 1 | 2 | 1 | 1 | 1 | 8 |
| Nohra, et al  2017[12] | 1 | 1 | 0 | 1 | 2 | 1 | 1 | 1 | 8 |
| Zhang,et al  2018[13] | 1 | 1 | 0 | 1 | 1 | 1 | 1 | 1 | 7 |
| Alejandro,et al, 2019[14] | 1 | 1 | 0 | 1 | 2 | 1 | 1 | 1 | 8 |
| Zhang,et al,  2019[15] | 1 | 1 | 0 | 1 | 2 | 1 | 1 | 1 | 8 |
| Salem,et al,2021[16] | 1 | 1 | 0 | 1 | 2 | 1 | 1 | 1 | 8 |
| Ryotaro,et al,2022[17] | 1 | 1 | 0 | 1 | 2 | 1 | 1 | 1 | 8 |
